# Concomitant empyema and peritonitis with Morganella morganii in an immunocompetent patient: A case report

**DOI:** 10.22088/cjim.12.2.232

**Published:** 2021-03

**Authors:** Mahnaz Amini, Mohammad Reza Motie, Saeid Amel Jamehdar, Mohammad Reza Kasraei, Mansoore Sobhani

**Affiliations:** 1 Department of Pulmonary and Critical Care Medicine, Lung Diseases Research Center, Faculty of Medicine, Mashhad University of Medical Sciences, Mashhad, Iran; 2 Department of Surgery, Surgical Oncology Research Center, Imam Reza Hospital, Faculty of Medicine, Mashhad University of Medical Sciences, Mashhad, Iran; 3 Antimicrobial Resistance Research Center, Avicenna Research Institute, Mashhad University of Medical Sciences, Mashhad, Iran; 4 Lung Diseases Research Center, Faculty of Medicine, Mashhad University of Medical Sciences, Mashhad, Iran

**Keywords:** Empyema, Peritonitis, Diaphragmatic defect, Morganella morganii, Pyopneumothorax

## Abstract

**Background:**

Peritoneal infection following pleural empyema is not a common occurrence. Concomitant pleural empyema and peritonitis have been described in the literature mostly in immunocompromised patients with different pathogenic mechanisms and a wide array of microorganisms. Here we report a case of concomitant pleural empyema and peritonitis with an unusual microorganism in an immunocompetent host.

**Case presentation:**

The patient is a 42-year-old man with a history of 2 weeks epigastric pain who had been referred for surgical consult after failure of outpatient medical therapy. Physical examination at emergency ward revealed generalized abdominal guarding, tenderness and rebound tenderness. On emergent laparotomy, the peritoneal cavity was full of malodor pus. All abdominal viscera were intact but there was a 2x2 centimeter defect in the top of left hemi-diaphragm. Pus originated from the left thoracic cavity and then drained to the peritoneal cavity. *Morganella morganii* grew in the culture of aspirated pleural fluid. After abdominal lavage and chest tube drainage and receiving 14 days course of parenteral antibiotics, the patient experienced marked clinical improvement. Punctual history taking revealed a history of pneumonia before the beginning of abdominal symptoms.

**Conclusion:**

In concomitant empyema and peritonitis in an immunocompetent patient, one should keep in mind the possibility of diaphragmatic defect and infection by unusual organisms like *M. morganii.*

Pleural infection following abdominal infection has been described in the literature but underlying mechanisms are poorly understood (1). Empyema is the most complicated form of para-pneumonic effusion. It is usually due to bacterial infection in the adjacent lung parenchyma and diagnosed by aspiration of pus or documentation of a positive culture in aspirated pleural fluid (2). Concomitant thoracic empyema and peritonitis with kinds of microorganisms have been reported. This is one of the first reports of community acquired infection with *M. morganii *in an immunocompetent host which affected both the pleural and peritoneal cavities (3). *M. morganii *is a gram negative, facultative anaeorobic rod, belonging to the enterobactreiaceae family. *M. morganii *has frequently been identified as the cause of urinary tract infection in immuocompromised hosts. Its spectrum of infection ranges from sepsis, pneumonia to spontaneous bacterial peritonitis. Its prevalence is really low especially in competent hosts (4). In this article we report a case of concomitant thoracic empyema and peritonitis with an unusual microorganism. 

## Case presentation

The patient is a 42 year old previously healthy man presented with history of 2 weeks abdominal pain. Pain was in the epigastric and left upper quadrant regions aggravated by eating, concomitant with nausea and 2 times vomiting. After a few days of outpatient medical therapy his abdominal pain became intolerable and he was referred to our medical center for surgical consult. The patient reported some fever and anorexia too. He did not have any known history of diabetes, chronic liver disease, corticosteroid use, immunodeficiency states, diarrheal disease or important surgical history. He was a chronic oral opium user. 

On admission it seemed he was ill with mild tachypnea (respiratory rate of 24 per minute) with an oral temperature of 38.0˚ centigrade. He had poor dental hygiene. Abdominal examination revealed generalized abdominal guarding, tenderness and rebound tenderness. Laboratory tests revealed leukocytosis of 11,500/fl with 75% neutrophil and normal serum biochemical tests. 

Midline laparotomy with diagnosis of acute abdomen which was done revealed malodor pus in the peritoneal cavity. The abdominal cavity was explored for any possible sites of visceral gangrene or perforation but all of the abdominal viscera were intact. With further exploration, a 2x2 cm defect in the left hemi-diaphragm with flow of thick pus from the top of the left pleural cavity to the peritoneal cavity was found. One thoracostomy tube was placed in the left hemi-thorax for drainage of empyema and abdominal cavity was closed after vigorous washing. Closure of diaphragmatic defect was postponed till the total control of abdominal and thoracic sepsis. Punctual history taking after operation revealed a prior history of pneumonia presented with fever, cough and left pleuritic chest pain. Then patient developed full blown signs of peritonitis. Culture of pus showed positive culture for *Morganella morganii*. Antibiotic susceptibility testing showed susceptibility to ampicillin, cefepime, cephtazidime, ciprofloxacin, co-trimoxazole, gentamycin, meropenem, and piperacillin- tazobactam. The organism showed no resistance to any antimicrobial disc. Blood and urine cultures were negative. Abdominal ultrasound was normal. 

Chest radiograph showed left hydropneumothorax with consolidation in the underlying lung (figure- 1). Chest radiograph showed clearance of fluid after 10 days of chest tube drainage but pneumothorax persisted (figure- 2). So the patient was scheduled for negative pressure appliance to chest tube for expansion of the underlying lung. Chest high resolution computed tomography (HRCT) after abdominal surgery showed infiltration and consolidation in left lower lobe along with pneumothorax and a small subpleural parenchymal cavity which can be proposed as the possible explanation of pyopneumothorax (figure- 3). 

**Figure-1:**
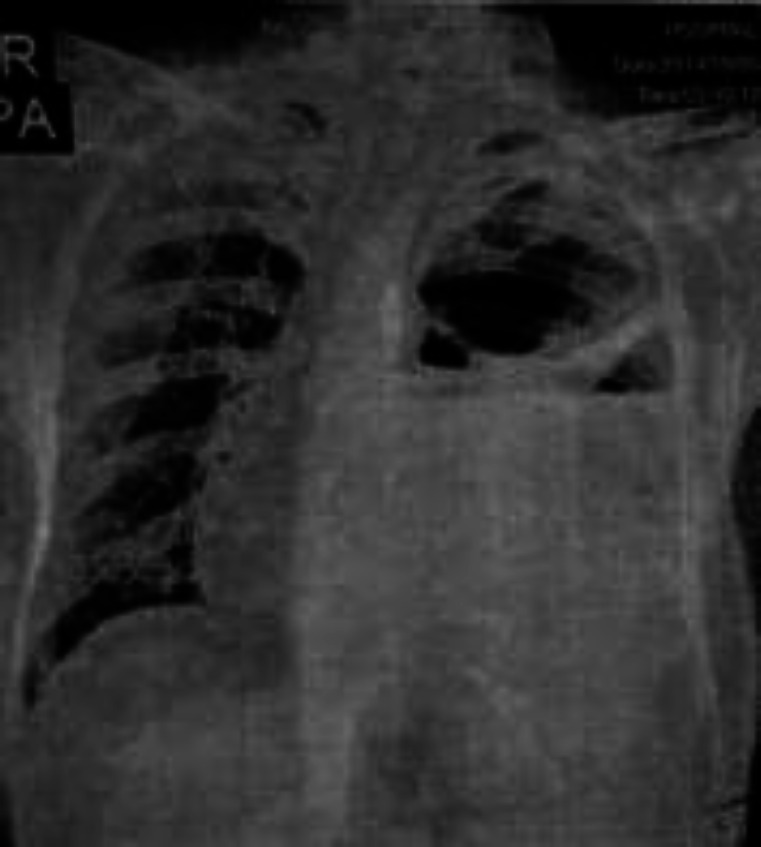
Chest radiograph before laparatomy and chest tube drainage revealing left sided hydropneumothorax

**Figure-2:**
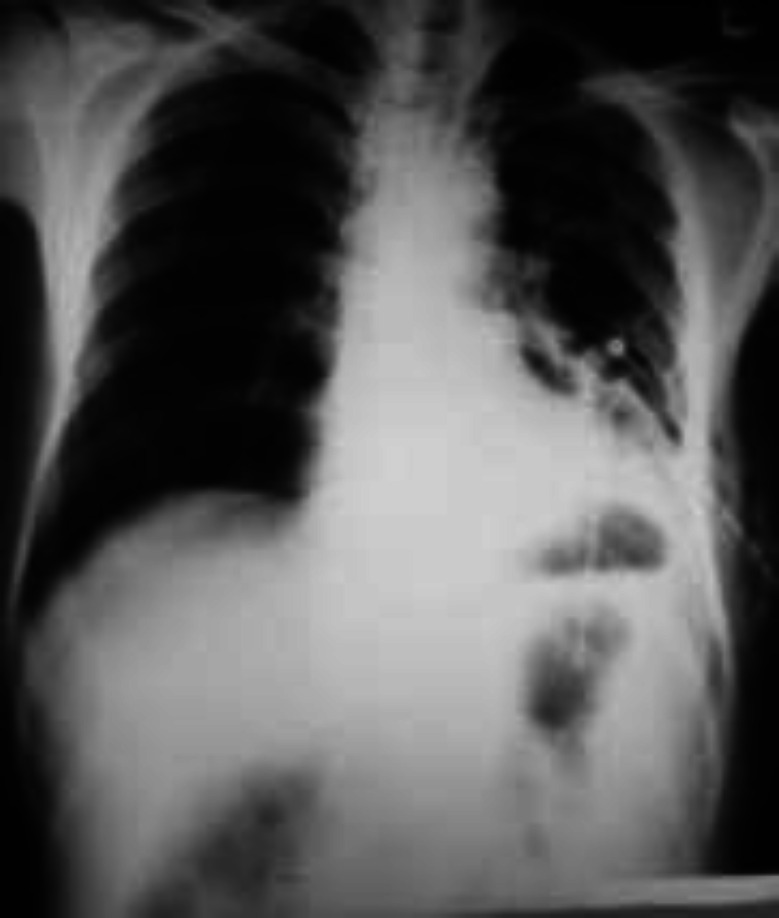
Chest radiograph after laparatomy revealing expanded left lung with chest tube in the posterior recess of the diaphragm

**Figure-3:**
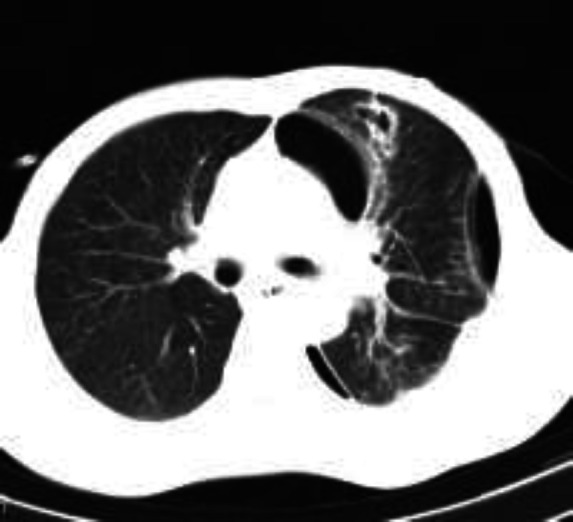
Lung HRCT after laparatomy and chest tube drainage revealing left sided hydropneumothorax. Arrow pointing at a peripheral cavity in the left lung parenchyma

Bronchoscopy at 11th day of surgery showed normal bronchial tree with pus coming out of the left main bronchus. Bronchial lavage fluid analysis showed acute inflammatory exudates with negative smear and culture for fast and slow growing microorganisms (including acid fast bacilli). 

The mainstays of treatment in the patient included peritoneal lavage and pleural drainage. Also he received a 14 days course of parenteral antibiotics (meropenem 1 gr IV q 8 h, ciprofloxacin 400 mg IV q12h and vancomycin 1 gr IV q 12 h). Patient showed marked improvement after control of thoracic sepsis. His leukocyte count was 5.300/fl at the time of discharge. 

## Discussion

Here we reviewed some of the reports of simultaneous empyema and peritonitis with *M. morganii* and some other pathogens. Most of these cases have occurred in the setting of severely ill septic patients or an underlying hepatic or peritoneal disease such as liver cirrhosis or peritoneal dialysis. 

Isobe et al. reported a case of concomitant peritonitis and thoracic empyema by *M. Morganii *in a cirrhotic patient (5). 

Cirrhotic patients with ascites have a propensity to develop spontaneous pleural empyema as well as simultaneous infection in peritoneal and pleural space. Cirrhotic patients usually have widened diaphragmatic pores with flow of ascites to pleural cavity and evolution of hydrothorax. This hydrothorax along with ascites could then be infected by bacterial flora of the intestinal tract and led to concomitant pleural empyema and ascites. This group of patients also showed some defects in the complement system (6). Some other organisms which were reported as the causes of simultaneous pleural and peritoneal infection in cirrhotic patients are as follows: *Pasteurella multocida* (7), *Listeria monocytogenes* and even along with pericarditis (8). 

Some other reports of this concomitant occurrence are in the setting of surgical patients. Some examples included subphrenic appendicitis (9), 12 cases of empyema after abdominal sepsis during 9 year (10) concluded that empyema after abdominal surgery needs evaluation for subphrenic abscess. subphrenic abscess (11) in a 75-year-old female *Bacteroides fragilis* and *E. coli* which CT showed a subphrenic abscess around the spleen, along with psoas abscess in a diabetic patient (12), following gastrointestinal intervention (13, 14). Ascitic and pleural fluid infection by listeria monocytogenes is uncommon. The association of spontaneous bacterial peritonitis and empyema caused by this microorganism has been seldom reported. A 61-year-old male with alcoholic cirrhosis and an upper right lobectomy for a lung cancer was consulted because of an exacerbation of dyspnea, abdominal pain and fever. Listeria-monocytogenes was isolated from ascitic and pleural fluids and blood cultures. He was successfully treated with ampicillin and a chest tube for drainage. There are some reports of simultaneous empyema and peritonitis in peritoneal dialysis patients. 

 In conclusion, this is a case of concomitant pleural empyema and peritonitis with *M. morganii *in an immunocompetent host. In the case of concomitant empyema and peritonitis in an immunocompetent patient, one should keep in mind the possibility of diaphragmatic defect and infection by unusual organisms like *M. morganii.* Accurate history taking is still an important tool in exploring the source of acute abdomen even in the modern era of medicine.

## References

[1] Tan PS, Badiei A, Fitzgerald DB, Kuok YJ, Lee YG (2019). Pleural empyema in a patient with a perinephric abscess and diaphragmatic defect. Respirol Case Rep.

[2] Yataco JC, Dweik RA (2005). Pleural effusions: evaluation and management. Cleve Clin J Med.

[3] Singh SA, Jeyasekharan DD, Jeyasekharan SS (2019). Concomitant primary peritonitis, septic shock and empyema thoracis in a young girl: a rare case report. Int Surg J.

[4] Liu H, Zhu J, Hu Q, Rao X (2016). Morganella Morganii, a non-negligent opportunistic pathogen. Int J Infect Dis.

[5] Isobe H, Motomura K, Kotou K (1994). Spontaneous bacterial empyema and peritonitis caused by Morganella morganii. J Clin Gasteroenterol.

[6] DeMeo AN, Andersen BR (1972). Defective chemotaxis associated with a serum inhibitor in cirrhotic patients. N Engl J Med.

[7] Fernández-Esparrach G, Mascaró J, Rota R, Valerio L (2010). Septicemia, peritonitis, and empyema due to Pasteurella multocida in a cirrhotic patient. Acta Gastroenterol Latinoam.

[8] Murray HW, Marks SJ (1977). Wiad L. Spontaneous bacterial empyema, pericarditis, and peritonitis in cirrhosis. Gastroenterology.

[9] Domaradzka-Wozniak A, Sawlewicz L (1962). A case of suppurative peritonitis and pleural empyema caused by Bacteroides Serpens during the course of therapy of cancer of the cervix uteri. Pol Tyg Lek.

[10] Ballantyne KC, Sethia B, Reece IJ, Davidson KG (1984). Empyema following intra-abdominal sepsis. Br J Surg.

[11] Okano A, Shibata M, Sato A (1992). A case of empyema with subphrenic abscess. Kansenshogaku Zasshi.

[12] Liu L, Goh ZW, Rhodes B (2013). Empyema and psoas abscess in a previously undiagnosed diabetic patient. N Z Med J.

[13] Sunder-Plassmann L (1989). Pleural empyema as a complication of gastrointestinal interventions. Langenbecks Arch Chir Suppl II Verh Dtsch Ges Chir.

[14] García-Olmo D1, Vázquez P, Cifuentes J, Capilla P, López-Fando J (1996). Postoperative gangrenous peritonitis after laparoscopic cholecystectomy: a new complication for a new technique. Surg Laparosc Endosc.

